# Community Participation and Empowerment in a Post-disaster Environment: Differences Tied to Age and Personal Networks of Social Support

**DOI:** 10.3389/fpsyg.2020.01802

**Published:** 2020-07-28

**Authors:** Ailed Daniela Marenco-Escuderos, Ignacio Ramos-Vidal, Jorge Enrique Palacio-Sañudo, Laura Isabel Rambal-Rivaldo

**Affiliations:** ^1^Grupo de Investigación PSICUS (Psicología, Cultura y Sociedad), Corporación Universitaria Reformada, Barranquilla, Colombia; ^2^Departamento de Psicología Social, Universidad de Sevilla, Seville, Spain; ^3^Grupo de Investigación CAVIDA, Universidad Pontificia Bolivariana, Montería, Colombia; ^4^Grupo de Investigaciones en Desarrollo Humano – GIDHUM, Universidad del Norte, Barranquilla, Colombia

**Keywords:** age, flood, gender, social participation, social support, personal network, disaster

## Abstract

In this article, an attempt was made to identify the level of community social participation according to age, gender, and the structural characteristics of the personal support networks in a population displaced by floods in the Colombian Caribbean. The research was based on a non-experimental methodology with an associative-relational strategy. An intentional non-probabilistic sample of 151 people affected by the winter wave in the south of the Department of Atlántico (Colombia) was selected. In total, the study included 42 males (27.8%) and 109 females (72.2%) participants, with an average age of 37.48 (±14, ranging from 18 to 80) and average relocation time of 21.79 months (±8.22, ranging from 5 to 36). The *Arizona Social Support Interview Schedule* (*ASSIS*) and Community Empowerment instruments were responded to with an instrument adapted from the *leadership competence* factor. The results show lower rates of intermediation in the older population, and the relationship between social participation and gender shows equally cohesive social support networks in men and women. This evidence is discussed to promote psychosocial interventions aimed to increase community engagement and empowerment of people that have experienced non-voluntary mobility processes.

## Introduction

Natural disasters produce mobility processes that affect large masses of people worldwide. Between 2007 and 2017, there was an average of 6,194 natural disasters in the world (United Nations Department of Economics and Social Affairs, 2017; Leiva-Bianchi et al., 2018), whereas in 2018, over 17 million people had to abandon their homes as a consequence of natural disasters [Internal Displacement Monitoring Centre (IDMC), 2019]. In the case of Colombia, natural disasters caused the displacement of 67,000 people, where at least 22,000 were affected by floods. Similarly, it has been shown that climate change combined with extreme poverty situations generates a series of psychosocial effects such as inequality and vulnerability, as well as poor management of the use of natural resources that combined increase the risk of displacement [Internal Displacement Monitoring Centre (IDMC), 2019].

However, it is worth noting that there is no integrating concept for the effects of natural disaster in people’s lives and their communities when in situations of vulnerability, natural disasters alter the everyday nature of the human being. Natural disasters alter the daily life of human beings, modify the physical and social space of communities, and together with other evolutionary and cultural processes, transform the personality and emotional ties of people who are impacted (Schiff, 2002; Thois, 2011; Leiva-Bianchi et al., 2018). In parallel, these environmental changes produce situations that allow people to develop their own capacities, pro-social behaviors, and to train coping strategies aimed at minimizing the consequences of disasters and strengthening the social fabric (Vollhardt, 2009). Negative situations generated by the interaction with the environment are a more evident social reality, whereby more individuals and geographical areas are at risk every year, especially due to factors such as population growth, increasing economic and social inequality, migration, and urban development in hazard-prone areas such as coastal regions (Crossett et al., 2004).

During the winter of 2010 and the beginning of 2011, the worst flooding in the last 40 years took place as a result of the climatic phenomenon known as “La Niña.” In the Department of Atlántico, 44,000 hectares were flooded, and 92,000 people had to be evacuated. Six municipalities in this department suffered the most severe flooding. Almost 100% of the region was affected with disconcertingly large material loss. In the process of post-disaster recovery, this scenario raised the need to rethink the role that the community plays in the decision-making, organization, and planning processes of its environment, but mediated specifically by the control and supervision of the community itself, which, in the end, is what suffers the most intense consequences of disasters (DRM – Disaster Risk Management).

Traditionally, the question has been asked about how much people in a community can actually contribute to the day-to-day political and administrative exercise. Likewise, compared to those who manage to contribute, how many manage have their concerns echoed within government provisions? Although we cannot deny that in one way or another community leaders, the representatives of civil society, are always present, the empirical evidence suggests that in recent decades there has been a reduction of social participation, civic engagement, and mutual support networks, factors that underscore the decline of social capital (SC) in modern societies (Putnam, 2000).

Community involvement and a willingness to invest time and resources in pro-social initiatives is one of the core values of SC as a multi-dimensional construct necessary for communities to achieve optimal levels of social welfare development (Cuthill, 2003). Along these lines, social participation strengthens social cohesion and contributes to keeping the members of the community united in the face of situations of adversity, this variable being a trigger for progress and sustainable development through the perspective of human intervention.

Participation and community involvement then contribute to the development of different modalities of relational SC. The networks of commitment or mutual support are based on the establishment of relations, and in this sense, they refer to the SC that can be presented in fewer than two modalities: *bonding* and *bridging*. The *bonding* type SC is based on norms of commitment and reciprocity that promote social cohesion, while the *bridging* type SC can be activated when collaboration requires entering into contact with external agents of the community-NGOs, formal institutions outside the community, among others, which diversify social networks and increase their heterogeneity (Poortinga, 2012).

In disaster management, community response is central to the extent that sometimes the absence of institutional resources forces the community itself to mobilize to cope with the effects of the disaster (Pandey and Okazaki, 2005; Chan et al., 2019; Zahnow et al., 2019). Similarly, thanks to social participation, people take part in decisions related to the environment and situations that affect them (Heller et al., 1984), which allows individuals to develop multiple leadership roles in social organizations and be central actors in the process of community change. According to Singer et al. (2002), through social participation, it is possible to strengthen competencies that allow communities to provide themselves with support to face the effects of crisis events. People who develop active social participation have positive attitudes, such as emotional maturity, self-confidence, persistence, among others, which encourage them to work on exploring lines of action that benefit their environment and contribute to their psychosocial well-being, predicated on a clear moral and social identity (Hart et al., 1998; Howells et al., 2013; Liu et al., 2018).

In this sense, the decision-making process and the degree of involvement people have in their own lives are defined as Community Empowerment (Rappaport, 1981). However, theoretically speaking, this is a systemic construct that can be studied from different perspectives: as a process or an outcome in itself (Zimmerman, 2000). Authors like Ohmer (2007) and Perkins and Zimmerman (1995) identified empowerment as a variable intrinsically related to community participation, among other things, because from the psychological point of view, when people participate to a greater extent in activities that concern their environment they begin to develop a feeling of control, dominion, and belonging over what happens there; in other words, they feel directly involved in the actions and consequences of each of the situations that arise in their community.

Some studies that analyze social participation in community contexts show the active involvement of the youth population in programs that work for the good of the communities, such as social volunteering and service actions (Flanagan et al., 1998; Flanagan, 2004; Dreyer and Guzmán, 2007). Accordingly, these results are supported by the empowering effect of social participation in the psychosocial adjustment of young people. Thus, it has been contrasted that the fact of feeling involved in the community and participating in it as an agent of improvement generates in the young person feelings of competence, self-esteem, and self-efficacy, which contributes significantly to greater satisfaction with life (Zimmerman, 2000; Moos, 2005; Palomar and Lanzagorta, 2005; Rodríguez et al., 2006; Smetana et al., 2006; Vieno et al., 2007; Buelga and Musitu, 2009). Because of these multiple contributions, the feeling of contributing to the life of the community, and to its constant improvement, is considered an important and effective element in community development.

Integration and community participation have been considered among the factors that have the greatest impact on social empowerment processes in the adult population. However, there are also aspects that encourage the processes of loss of power and involvement in decision-making and problem solving in adults, such as the negative social representation of adulthood and old age as well as the models to be followed in relation to the use of power and hierarchies in adulthood (Lacub and Arias, 2010; Sánchez-Vidal, 2017). Although there is evidence that the recognition of social value and utility in adults has a positive impact on psychological functioning and also decreases the risk of mortality (Ekerdt et al., 1985), what we are observing in vulnerable communities is the prevalence of the young population in the areas of appropriation and leadership, a point that calls us to discuss the contribution of youth, adults, and seniors in community development.

For example, Arias et al. (2009) found that adults consider having good family and social relationships to be key aspects for their lives, since they promote greater and better levels of participation, integration, and social support, variables that are related to higher levels of life satisfaction. Other studies show that adults can be active agents who are satisfied with their lives and possess multiple personal strengths that allow them to improve the quality of life in their communities (Carstensen et al., 2000; Lacey et al., 2006; Wood et al., 2007; Fernández, 2015). Colombia is a country marked by recent revolutions in social, environmental, and political customs, which have had the effect of transforming the people’s levels of participation in the decision-making processes that affect them (Arias-Cardona and Alvarado, 2015).

On the other hand, in addition to age, gender should also be considered when assessing the background and consequences of participation processes. Gender is an important factor when it comes to understanding the relation between social support and different variables. Empirical evidence shows an important role as a moderator in the association between post-traumatic stress disorder (PTSD) and traumatic effects in natural disaster survivors (Bonanno et al., 2010; Han et al., 2019). In a more specific manner, it has been proven that women tend to be more receptive to the positive, protective effects of social support in diverse situations, which have an effect on mental health (Andrews et al., 2003; Anhern et al., 2004).

Various investigations show that the structure of social support networks differs according to gender (Maccoby and Jacklin, 1974; Troll, 1986). Women seem to have larger networks, with greater compositional diversity, who fulfill a greater variety of functions than men, whose networks, in contrast, tend to be smaller and the provision of social support tends to be concentrated in the spouse (female partner) (Babchuk, 1979; Campbell, 1980; Veroff et al., 1981; Longino and Lipman, 1983).

A recent study aimed at identifying the effects of forced displacement on the structure of personal networks and on community participation processes identified that women presented a more cohesive relational context (denser links) than men, something that in practice reduces the possibilities of establishing relations with members outside the social circle of the collaborators (Ramos Vidal, 2018). This phenomenon, to some extent, is explained by the incompatibility between the two modalities of SC mentioned above. This supposes that when the networks are very dense (*bonding* type) this withdrawal of relations makes it difficult for people to diversify their sources of social support (which limits the options to access the *bridging* type). On the contrary, in the levels of centralization and intermediation, higher and more significant values were found in men, which indicates that they have a greater diversity of their networks, being actors with greater facility to establish relations with subjects that belong to other social circles, something that facilitates access to different resources of social support and to subjects that form part of groups of greater social status (Lee, 2017).

Whereas SC in its relational aspect, i.e., considering the various resources that social support networks provide to subjects, and in its pro-social aspect, i.e., showing that community involvement allows affected persons to overcome adversities that occur as a result of natural disasters (i.e., Ganapati, 2012; Rahill et al., 2014), the main objective of this study is to examine both phenomena in a sample of affected people who suffered the effects of the winter wave. Therefore, this research aims to identify the relationship between participation and community empowerment across the variables of age, gender, and the structural characteristics of the personal networks of a group of people affected by natural disasters on the northern coast of Colombia.

Although the main objective of this investigation does not center in analyzing the individual impact that derivates from the flooding, it is convenient to highlight some of the main individual consequences that are a direct product of this type of disaster. Along this line, Alderman et al. (2012) examined through a systematic review, the health impact that floods have in the general population. The analysis of 35 epidemiological studies shows the negative effect floods may have, both short-term and long-term. Among the short-term effects, the study shows that during the year following the flood, the risk of generation of epidemics like Hepatitis E, Swamp fever, and gastrointestinal diseases rises notoriously. The study shows as well that 2 years following the flood, a significant proportion of survivors (between 8.6 and 53% depending on the study and relevance of the flood) develops numerous psychological disorders, such as PTSD, anxiety, or depression. Other investigations, in addition to emphasizing the psychological impact suffered by the affected population, display the effect that floods generate in the individual’s relationship with their families, communities, and interpersonal relationships. The study developed by Carroll et al. (2009) brought out that besides the material loses, the affected population suffers from feelings of alienation, difficulty to identify themselves with the context they are surrounded by, and self-control loss in relation to their environment. All of these effects are inversely-related to the population’s mental health.

## Methodology

### Design

The research was based on a non-experimental methodology with an associative-relational strategy (Ato et al., 2013), which sought to determine the possible relationship between social participation and empowerment to subsequently evaluate the differences of these “main” variables in accordance to gender, sex, and structural characteristics of their personal networks.

### Participants

The 2011 rainy season affected six towns in the south of the “Atlántico (Colombia)” department. In the context of the study, the biggest impact produced by the floods was the displacement of large masses of population. Even though, material losses, damages to housing, and private property are constant, substantial long-term effects in these settings, it is usually reflected in the process of forced displacement provoked by them. In the case of the “Atlántico” department, there was not a single fatal incident; however, more than 90,000 people lost their houses and were forced to be displaced. During the initial moments after the catastrophe, the affected people had to be temporarily relocated to safe zones. Afterwards, the population was relocated in residential communities promoted by the state in order to attend victims that were in a more vulnerable situation. Therefore, in this research, the displacement phenomenon caused by floods is examined as an articulating axle that allows us to comprehend (a) the characteristics and traits of communities where participants of the study reside and (b) the psychosocial effects experienced by the affected population.

For this study the selected town had 200 affected families and the total of this population was relocated to new houses, built by the city hall, located on the outskirts of town, in a non-inhabited terrain about 1 km away from their previous residencies; this whole relocation process was concluded in 2017. An intentional non-probabilistic sample of 151 people was selected, seeking representation of at least one member of each affected family. This methodological selection contributes to having a deeper knowledge of the effects at an individual, family, and communitarian level, owed to forced displacement generated by the floods. Something recommendable keeping in mind the negative effects and feelings of loss and alienation associated to this type of phenomenon (Hawkins and Maurer, 2011).

This work was developed after the process of permission from the Ethical Commitee of the Universidad del Norte Foundation, ensuring the fulfillment of the ethical principles of investigation on a national and international level. In accordance to the established in the resolution 8430 of 1993, chapter, article 11, issued by the “*Ministerio de Salud y Protección Social*” (Ministry of Health and Social Protection) of Colombia, this research is considered as a low risk research to its participants because it collects information that contains sensitive aspects of the subjects behavior, particularly personal feelings. Besides, the objective population, being affected by a natural disaster, immediately suggests a state of social vulnerability. The participants in this study were contacted directly, the goals and objectives of the study were presented clearly, and a detailed description of their rights was carried out. Subsequently, the subjects proceeded to sign a consent waiver, where it clearly stated that agreeing to join the study did not imply obligation to remain and they could leave whenever they pleased.

The sample comprises 42 men (27.8%) and 109 women (72.2%). The gender imbalance of the participants is explained by the fact that most of the people benefiting from the relocation and new housing by the local government had to meet different criteria and the local administration prioritized those who met requirements such as head of household, single mother, or caretaker of other dependents. The average age of the participants is 37.48 years (±14, Range 18–80), and the average relocation time in the destination community was 21.79 months (±8.22, Range 5–36). Most of them were in a stable marital relationship, either married (*n* = 18, 11.9%) or in a civil union (*n* = 106, 70.2%), and their level of education was low, most of them with incomplete basic studies (*n* = 41, 27.2%), followed by those with incomplete (*n* = 39, 25.8%), or complete (*n* = 26, 17.2%), intermediate studies. Likewise, 8.6% (*n* = 13) did not complete their basic studies, while 1.3 (*n* = 2) did not complete them at the technical level, but 7.3% of the sample (*n* = 11) did.

### Instruments

In the following, the instruments used for the measurement of each one of the main variables are described:

#### Social Support – Community Participation

The *Arizona Social Support Interview Schedule* (*ASSIS*) (Barrera, 1980) was used to measure this variable. It was adapted to evaluate the frequency and types of social support that each component of the personal network (alter) provides to the interviewee (ego). The ASSIS consists of creating a list of the names of people who offer social support both in term of perceived and actual support received up to 1 month prior to the interview. The interview collects information on six specific forms of social support that appear in the literature: (a) *expression of personal issues*, (b) *material assistance*, (c) *advice*, (d) *positive feedback*, (e) *physical assistance*, and (f) *social participation*. Each one of these forms is measured by the way it is perceived and received. For the purpose of this study, the version adapted by Maya and Holgado (2005) was used, which presents a 0.88 reliability test-retest after a 3-day evaluation (López et al., 2007) and the values obtained in the last of the forms of support were specifically taken as follows. Social participation is measured in an escalating manner with 12 items; in this study the specific subscale had moderate reliability (*a* = 0.69). The participants must answer four questions reporting the number of people on their network that would apply to each case, e.g., *how many people would they gather with to have fun or relax?* Or *in the last month, with how many of those people have they genuinely gathered with?*

#### Characterization of Personal Networks

In order to identify the properties of the participants’ personal networks, each participant (called ego) had to nominate among their contacts the people (called alteri) who provide them with each of the six types of social support that are evaluated through ASSIS. It was decided to establish a limit of 20 actors for the size of the participants’ personal networks, since it is suggested that this size is sufficient to capture the different social circles that shape the structure of the personal networks (McCarty, 2002), and at the same time a fixed number of actors is established in order to be able to make comparisons between the structural parameters of the networks. Once the network contacts have been identified, ego should indicate what relationship each pair of alteri has with each other in a dichotomous response format, where 0 indicates that they do not know each other and 1 indicates that the alteri know each other.

#### Community Empowerment

This variable was evaluated with an adapted instrument derived from the factor “leadership competence” included in the Sociopolitical Control Scale designed by Zimmerman and Zahniser (1991) and revised by Speer and Peterson (2000). The original instrument allows the evaluation of leadership capabilities of anyone, around activities that pointed toward improving environmental conditions, quality of life in community contexts, and whether it can be managed by social groups or not. The leadership skills subscale consists of eight items with different response options on a Likert-type scale (from 3 to 7 options), but the adaptation for Colombian population includes five items: (a) *I am often the leader in the groups that I am part of*, (b) *I prefer to be a leader rather than a follower*, (c) *I can organize people to do positive things in the community*, (d) *I enjoy participating in political affairs because I have a lot to say on issues that affect my community*, and (e) *People like me are qualified to participate in political activity and in the decision-making process of our country*, all of them organized on a Likert-type scale of four options (1 = total agreement, 4 = total disagreement). Its use on a Colombian vulnerable population showed an acceptable level of Cronbach alpha (*a* = 0.73) (Ramos-Vidal, 2014) in this study it presented *a* = 0.75.

On this study, the concept of community is assumed and comprehends a geographical and relational vision. Thus, community (both in the physical and geographical context where participants reside) is the combination of social relations that individuals establish with other residents in the same context (i.e., Heller, 1989).

### Data Processing and Analysis

The data about the personal network members and the relationships that link each dyad was transferred to a 1-mode symmetric adjacency matrix (Wasserman and Faust, 1994). Subsequently, cohesion indicators of personal networks were calculated to describe their structural variability (McCarty, 2002; Molina, 2005; Bidart et al., 2018). The information collected in each matrix was processed with the UCINET 6.3 software (Borgatti et al., 2002), which was also used to calculate the structural indicators of personal networks. The indicators evaluated are the average value of the intermediation (*Betweeness*), the *geodesic* distance, and the density (*Density*). As Wasserman and Faust (1994, p. 188) indicates, *“Interactions between two nonadjacent actors might depend on the other actors in the set of actors, especially the actors who lie on the paths between the two. These “other actors” potentially might have some control over the interactions between the two nonadjacent actors. Consider now whether a particular actor might be able to control interactions between pairs of “other actors” in the network.”* […] The average betweenness value is calculated by adding the centrality of intermediation of each actor and this result is divided by the number of actors that make up the personal network, 20 in this case. *A shortest path, or geodesic distance, between two nodes in a graph is a path with the minimum number of edges* […] *The density of a directed graph is equal to the proportion of arcs present in the network. It is calculated as the number of arcs, L, divided by the possible number of arcs* (Wasserman and Faust, 1994, p. 129).

The NETDRAW application that is part of UCINET was used to carry out the visualization of networks. Then, a cluster analysis was performed following the K-means procedure (Dubes and Jain, 1988) to identify the typologies of participants according to age and the level of global intermediation of the personal network, in order to compare the level of perceived and received participation based on the conglomerate level of belonging of the participants afterwards. According to Luke (2005, p. 196), this analysis is particularly useful in community research because it allows for the establishment of participant profiles that are useful for segmenting the population into evaluation and intervention processes. Finally, non-parametric tests were carried out (χ^2^) using the Kruskal-Wallis test as a contrast statistic and the initial number of cases as a grouping variable (McKight and Najab, 2010).

## Results

To initiate the results report, we proceeded to calculate the correlation between the main variables was calculated: social participation and community empowerment, which were not significant (*r* = 0.387; *p* < 0.071) Subsequently, the clusters that can present discriminant validity over the networks of all the cases evaluated. This started with the cluster analysis using as grouping variables the age of the participants and the intermediation of the networks. The procedure has converged in four iterations and the minimum distance between the initial centers is 31,056 (see Table 1).

**Table 1:** Final centers of the conglomerates.

Grouping variables	Conglomerates		
	1	2	3	(*n* = 9; 5.9%)	(*n* = 61; 40.3%)
(*n* = 81; 53.6%)	Age	69	47
27	Intermediation	0.52	0.78	1.37

Source: own elaboration.

**Figure 1:**
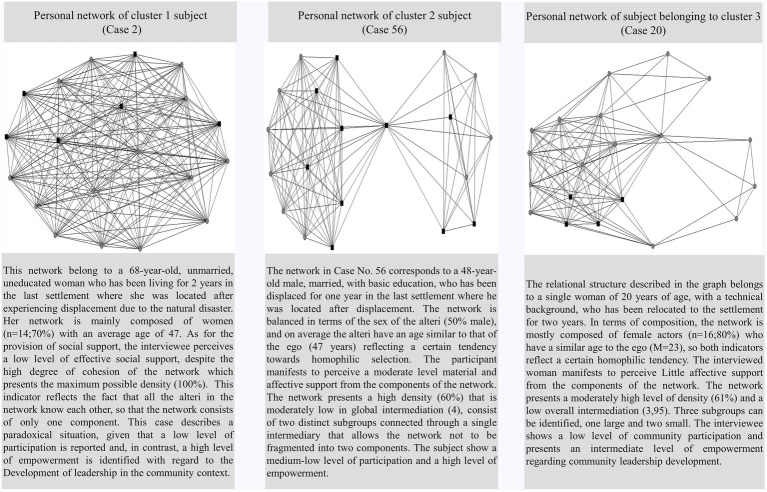
Example of a network per cluster.

This analysis suggests that older people have lower rates of intermediation, possibly because their networks are comparatively denser than those of younger people. Figure 1 presents the graphs of each cluster and provides a characterization of each case.

In line with previous studies (i.e., Van Tilburg, 1998; Phillipson et al., 2001), it can be argued that older people tend to have more cohesive networks and, at the same time, tend to concentrate sources of social support on high-intensity relationships with family and friends. Table 2 is updated with the descriptive and correlational statistics between all the variables studied (see Table 2).

**Table 2:** Descriptive statistics, reliability of the scales, and correlations between the study variables.

Variables	M	SD	1	2	3	4	5	6
1. Age	37.48	14	-	−0.177^*^	−0.199^*^	0.201^*^	−0.184^*^	0.096
2. Betweenness	1.08	1.55	-	-	0.884^**^	−0.805^**^	0.033	0.085
3. Geodesic distance	1.09	0.14	-	-	-	−0.912^**^	−0.011	0.019
4. Density	0.89	0.18	-	-	-	-	0.033	−0.062
5. Social participation	7.56	6.5	-	-	-	-	-	0.071
6. Empowerment	11.64	4.2	-	-	-	-	-	-

Source: own elaboration.

* *p* < 0.05;

** *p* < 0.001 (two-tailed).

Taking into account that the data did not meet the criteria of normality and homoscedasticity, we proceeded with the non-parametric Kruskal-Wallis analysis to identify whether there are significant differences in the three clusters detected as a function of the level of empowerment, using the values of the full ordinal scale for this analysis. This analysis did not show significant values, even with the time of relocation in the target community, but it did show significant values as a function of the age of the alters (*χ*^2^ = 56,214; *p* = 0.000), a situation that can be considered normal considering the principle of homophily (McPherson et al., 2001), according to which people tend to establish substantive relationships with people of a similar age (Lozares et al., 2014).

On the other hand, and taking into consideration classic studies on social support in adult men and women, the data suggest that women – majority in the study sample – have equally cohesive social support networks as men, something that contradicts the research background (i.e., Antonucci and Akiyama, 1987). This result is interesting because it seems to indicate that the living conditions fostered by the situation of displacement seem to eliminate the aforementioned differences, so that life experience becomes similar relational processes that lessen gender differences. To extend this analysis, all forms of social support (received and perceived) were compared with the gender of the participants. Table 3 shows, through the calculation of the Mann-Whitney U, the significant differences found. This analysis shows that men report better forms of perceived support such as material help, receiving advice when needed, and show a higher level of social participation.

**Table 3:** Comparison of social support according to gender.

Variables	U	Z	*p*	*r*	>MR^a^
Personal feelings	2218.5	−0.650	0.516	-	-
Material support	1979	−2.100	0.036^*^	0.17	Man
Council	1841	−2.036	0.002^*^	0.16	Man
Positive feedback	2029.5	−1.371	0.170	-	-
Physical assistance	2,217	−0.430	0.667	-	-
Social participation	1815	−2.326	0.020^*^	0.18	Man

Source: own elaboration.

a Middle range. *p < 0.05.

The analysis of the effect size of the differences that were significant shows us a small power so the results must be taken with moderation. However, it highlights that in all the forms of support it is the men who show a greater medium range, that is, they are the ones who develop more pro-social behaviors and have more social support in their community, referred in this case to the reception of material help and advice for decision-making.

Finally, we proceeded to calculate the possible existence of differences between clusters versus the participants’ perceived level of social participation (see Table 4). The most interesting result is that there are significant differences identified through the Kruskal-Wallis test between the three profiles and social participation measured as high, medium, or low.

**Table 4:** Comparison of social participation by conglomerate.

	Kruskal-Wallis				
	Conglomerate	*X¯*	*X*^2^	*p*	MR^a^
Perceived social participation	1	9	6.334	0.042^*^	56.44
	2	61	-	-	70.39
	3	81	-	-	82.40
	Total	151			
Social participation received	1	9	4.849	0.089	60.00
	2	61	-	-	72.43
	3	81	-	-	80.47
	Total	151			

Source: own elaboration.

* *p* < 0.005.

a Middle range.

After finding a significant difference between perceived social participation and personal network cluster groups, *post hoc* analysis was performed and no significant differences according to group pairs were identified. The following section discusses the results of the research.

## Discussion

The main purpose of this work is to identify the level of community social participation according to age and gender in the population displaced by floods in the Colombian Caribbean, several years after the event, and to observe the structural properties of the participants’ personal networks. First of all, it is surprising that in our data analysis there is no sign of relationship between social participation and community empowerment, an aspect that contradicts the empirical evidence deduced from the variables. However, although participation and the intrapersonal component of empowerment maintain a reciprocal relationship that in some cases makes these community processes appear in a transversal manner (Speer and Hughey, 1995; Itzhaky and York, 2003), there are cases in which the need to first establish optimum levels of participation so that empowerment can subsequently take place is justified (Christens et al., 2011). It is likely that the latter will occur in the population studied, and that community participation will be established in an isolated manner, for which reason strong processes of community empowerment have not yet been established to complement the process of social empowerment along with the characteristics of participation itself.

On the other hand, the lower intermediation rates in the older population are due to their more dense networks compared to the networks of the young. In this connection, previous studies, such as those developed by Van Tilburg (1998) and Phillipson et al. (2001), indicate that older people have more cohesive networks consisting mostly of family and friends, which may be an indicator that personal networks of adults are still organized in very dense or closed structures that reflect difficult social and environmental contexts, in which people support each other on a daily basis to survive in the face of adversity (Lomnitz, 1975; Sanandres et al., 2014). This phenomenon can also be explained by the fact that as the life cycle advances, people tend to withdraw their relationships into their closest contacts (Seeman and Berkman, 1988).

On the other hand, and as a consequence of our results, some studies of social participation in relation to age show the active involvement of the youth population in cooperation networks with solidarity and civic objectives in their communities (Flanagan et al., 1998; Flanagan, 2004; Dreyer and Guzmán, 2007). In this sense, the result obtained can be justified on the basis of studies that suggest that the new generations of young people are more active carrying out modalities of social participation from the community and from the everyday, instead of developing such activities from the traditional structures of socio-political participation (Harris et al., 2010). This phenomenon can also be approached from the point of view of the real inclusion of the young population as full citizens (Smith et al., 2005). This implies that participation is an instrumental mechanism through which young people guarantee their own inclusion in the social context, something that in the case of the study population is particularly relevant insofar as mobility processes often involve phenomena such as uprooting and even disempowerment that can be mitigated through participation (Lardier et al., 2019).

Likewise, critical situations, while they can be stressful and difficult to manage for some affected people, can also empower processes and skills for other participants in the community context, generating responses such as strengthening social organizations, increasing organizational capacity and community preparedness to deal with environmental disasters (Mavhura et al., 2017). In addition, community participation is energized, which in turn increases cohesion and the *bonding* type of SC that can be observed to some extent in these results. As a future challenge, it is necessary that public agencies and private organizations deal in parallel with the people and communities that have suffered this type of situation to amplify and diversify their networks (both from the individual and community levels).

A striking point is the relationship between social participation and gender, where it is evident that social support networks are equally cohesive in men and women. This situation to some extent contradicts the findings of classic studies on the structural evaluation of social support (i.e., Antonucci and Akiyama, 1987; Seeman and Berkman, 1988; Neff and Karney, 2005). This result is of interest since it seems that the living conditions of the sample, in this case marked by mobility processes in rural contexts, can eliminate the aforementioned differences, so that life experience becomes similar to relational processes that reduce the effect of gender differences.

Participation generates opportunities to socialize and, in this case, also to diversify interpersonal relationships and sources of social support (i.e., Mollenhorst et al., 2008), aspects that have been recognized as precursors to the emergence of positive resources especially in vulnerable populations. Furthermore, they acquire importance through the enrichment of the understanding of how the relationships created after the tragedy that breaks with the “everyday” can help to re-establish, re-organize, and re-structure new spaces of participation to improve the living conditions of the community. Normally, in disaster situations, human beings tend to manifest fight or flight responses, where feelings of initiative do not usually appear, and it is preferred to opt for the path of abandonment of space or exodus to achieve a new life (Barrios, 2013). In this way, participation produces positive effects of a multilevel nature – individual, family, community, and society – connected to empowerment, the achievement of social justice, the strengthening of grassroots organizations, and associative movements, and in this particular case, the resilience of communities that have suffered serious life stressors (Sherrieb et al., 2010; Aldrich and Meyer, 2015).

As it was mentioned on the utilized research design and with the available information, is not possible to know if the relocation process brought out the urgency to create new social bonds that, at the same time, would affect the structure and composition of personal networks. However, previous studies that analyze the adaptation process experienced by displaced communities in their destination context repeatedly suggest that this population find several difficulties to rebuild their networks of social support with residents in their receiving communities. In the case of Colombia, it has been documented that the displaced population have frequently suffered defamation and discrimination by the inhabitants the destination context. This has contributed to the generation of conflict between groups and social polarization episodes. Both processes have an effect in the relational strategy of the displaced individuals, who tend to maintain relationships with only with people whom are found in the same situation, which makes frequent the activation of relationships with people of the immediate environment (this favors bonding-type SC), but simultaneously, restrains the activation of relationship with the population from the destination context who cannot be categorized as displaced population (this makes the creation of bridging-type SC difficult) (i.e., Kaniasty and Norris, 1993). This phenomenon may somewhat explain that such relational strategy contributes indirectly to the maintenance of the social vulnerability situation that affects the displaced population as a whole. Not depending on a withdrawal of interpersonal relationships, precedents suggest that to suffer from this type of disaster, is associated with the loss of social support (i.e., Kaniasty and Norris, 1993).

Being able to understand the dynamics of social processes and related variables turn into a positive tool to create intervention dynamics, whose main objective is to guarantee a successful positive adaptation of vulnerable populations. For that matter, it is important to understand the effects that natural disasters have in people and their communities because it can be useful in order to have only one conceptual line that articulates processes and consequences of catastrophes, which can contribute to the purpose of improving the quality of life of the people in vulnerable situations (Leiva-Bianchi et al., 2018). Different authors like Guilaran et al. (2018) and Kaniasty and Norris (2009) state that there are three different sides, after a disaster that controls the effect of social support in victims. (1) receipt of actual assistance, (2) perception of availability of support, and (3) integration in a network of caring individuals. Following these sides, the relationship between social support and the protective effects that were created start to decline. This supports the idea that communities will take longer to overcome the hindering conditions that they find themselves in after the disaster, from an economical, physical, social, and organizational point of view.

Furthermore, these results show the potential that network analysis and forms of social support bring to understanding complex social processes and the increase in positive levels of social evolution in affected communities in the rural context and in the Colombian Caribbean. The purpose of this research was to analyze the community participation processes within the dynamics of structuring and coexistence in society of a type of vulnerable population that has suffered the consequences of disasters. However, this evaluation would be enriched by the inclusion of other variables such as the sense of community, community preparedness to respond to this type of situation, and the construction of organizational capacity that makes it possible to face adverse situations such as the one described in this paper (Gil-Rivas and Kilmer, 2016). Similarly, it can be indicated that the limitations of this study are centered on the difficulty of accessing a more representative sample in accordance with the inclusion criteria managed, which translates into a reduced discriminating power in the analysis of the variables. In addition, a discriminant data analysis could be included in the absorption/response rate of the participants, that is, how many of those who were contacted responded to the study and classified it by gender, age, people with whom they live, among others.

Notwithstanding the objective of the research is not to examine, from a comparative point of view, the likely similarities and differences on the relational patterns of the individuals who have suffered from the effects of floods against those who have not, it is imperative to specify some of the aspects that may contribute to the understanding of this phenomenon from a relational perspective. First, the relocation process caused by the floods produces damages to the structure of social supper that surrounds the individuals. Secondly, the affected population faces a traumatic experience that includes the loss of material belongings and identification symbols from their communities, which reverberates on the perception that the individual has over the environment inhabited. Thirdly, the displacement of population provokes (usually abruptly) the rupture of interpersonal relationships in different intensities (Kaniasty and Norris, 1993; Carroll et al., 2009). Keeping in consideration the exposed elements, it seems plausible to propose that the structure, the composition, and the properties of the social support networks may be different from the population or communities that have not suffered the effect of natural disasters such as the one reported in this work.

Finally, for further studies, it is recommended to include in the model possible differences in the forms of support and in the composition of personal networks according to different socio-demographic variables. In order to better appreciate the changes in personal networks before and after the winter wave, a longitudinal design is suggested that takes into account the details of the composition of everyday relationships. It would be interesting to carry out a comparative research on a retrospective level that allows the evaluation of the networks before and after being in a situation of vulnerability with the objective of identifying if there were or not negative effects from the disaster directly on personal relationships against other populations that have suffered similar disaster situations in other regions of the country. Furthermore, that also requires the use of memory by the participants to describe the old personal networks and their characteristics as opposed to the community empowerment before and after the crisis that led them to a state of greater vulnerability. As an important topic, it must be taken into consideration that on the interventions in populations who are victims of disasters, the community empowerment component must prevail as well as the strategies to strengthen social network.

## Data Availability Statement

All datasets presented in this study are included in the article/supplementary material.

## Ethics Statement

The studies involving human participants were reviewed and approved by Comité de ética Universidad del Norte. The patients/participants provided their written informed consent to participate in this study.

## Author Contributions

AM-E, IR-V, and JP-S contributed conception and design of the study. AM-E organized the database. IR-V performed the statistical analysis. AM-E and LR-R wrote the first draft of the manuscript. All authors contributed to the article and approved the submitted version.

### Conflict of Interest

The authors declare that the research was conducted in the absence of any commercial or financial relationships that could be construed as a potential conflict of interest.
